# Adaptation and validation of the quality of life assessment of the Cambridge pulmonary hypertension outcome review (CAMPHOR) for Brazil

**DOI:** 10.1186/s41687-020-00209-6

**Published:** 2020-06-05

**Authors:** Ricardo Amorim Corrêa, Monica Corso Pereira, Mariana Ferreira Bizzi, Rafael W. R. de Oliveira, Camila Farnese Rezende, Bruna Cristina Marabita Tavares de Oliveira, Alice Heaney, Stephen P. McKenna, Antonio Ribeiro-Oliveira

**Affiliations:** 1 Department of Internal Medicine, Federal University of Minas Gerais, Belo Horizonte, Minas Gerais Brazil; 2 Department of Internal Medicine, University of Campinas, Sao Paulo, Brazil; 3 Galen Research Ltd, Manchester, UK

**Keywords:** Pulmonary hypertension, Quality of life, Questionnaire, Patient outcome

## Abstract

**Background:**

Pulmonary Hypertension (PH) impacts negatively on patients’ health-related quality of life (HRQoL). The Cambridge Pulmonary Hypertension Outcome Review (CAMPHOR) was the first PH-specific and validated instrument for use in different languages worldwide. This report describes the adaptation and psychometric validation of the CAMPHOR into Brazilian Portuguese language.

**Methods:**

The translation and validation process included a bilingual and lay panel translation; cognitive debriefing interviews; psychometric testing in two repeated times assessing internal consistency, reproducibility and validity of the questionnaire. The Nottingham Health Profile (NHP) questionnaire was used as a comparator to test for convergent validity.

**Results:**

The translation captured the same concepts as the English questionnaire and produced a comprehensive instrument in a Brazilian-Portuguese version expressing common, natural language. The psychometric evaluation involved 102 patients (48.8 ± 14.5 years, 80,4% female]. Cronbach’s alpha coefficients were above 0.9 on all three CAMPHOR scales. There was excellent test-retest reliability (coefficients above 0.85 on all scales). CAMPHOR Symptoms scale and Activities scale correlated highly with Physical Mobility section and CAMPHOR QoL scale was strongly associated with the Emotional Reactions and Social Isolation sections of NHP. There was a significant association between gender and perceived general health (*p* < 0.05). There were significant differences in CAMPHOR scale scores between patients who differed according to their perceived disease severity and general health.

**Conclusions:**

The present CAMPHOR version demonstrated good psychometric properties and provides a reliable instrument for assessing HRQL and QoL in Brazilian PH patients, addressing patients’ perspective of their illness in a comprehensive way.

## Introduction

The term pulmonary hypertension (PH) encompasses a variety of clinical disorders characterized by an increase of pulmonary artery pressure and pulmonary vascular resistance that leads to exercise capacity limitation due to right heart failure and ultimately death [1]. International guidelines have classified PH into five groups based on similarities between the pathobiology of specific etiologies. Thus, PH has been classified as precapillary (Groups 1, 3, 4 and 5) or postcapillary (Groups 2 and eventually 5) [1]. Group 1 pulmonary arterial hypertension (PAH), and a subgroup of Group 4, chronic thromboembolic pulmonary hypertension (CPTEH), are currently susceptible to specific therapeutic interventions developed in the last decades. Most patients with PH present with non-specific symptoms such as shortness of breath, exertional dyspnea, chest pain, fatigue or syncope that can progress slowly over time, leading to delays seeking a medical diagnosis [2].

Advances that have been achieved in the understanding of the mechanisms and pathobiology of PH has led to the development of drugs and newer strategies directed at specific targets, which have resulted in improvement in many outcomes. World Health Organization functional class (WHO FC), six-minute walk test (6MWT), biomarkers of cardiac function (BNP and NT-proBNP), hemodynamic parameters (right atrial pressure, mixed venous saturation, cardiac output and cardiac index, and pulmonary vascular resistance (obtained through cardiac right catheterization) have been extensively studied in pivotal clinical trials, and national and international PH registries. However, despite these advances, there is substantial heterogeneity in terms of morbidity and survival among those conditions even in the same group [1, 3–5].

As a result of these advances, patients have shown significant improvement on their generic health-related quality of life (HRQoL), morbidity and mortality rates. It has been acknowledged that it is important to take into account patients’ perspectives while caring for these individuals. Generic HRQoL questionnaires were first used to address this issue showing some prognostic utility in PH [6–10]. However, a poor correlation between these tools on some domains of HRQoL has been reported, pointing out to the need for disease-PH-specific instruments [8, 10]. To bridge this gap, specific questionnaires for PH have been developed [11]. However, it is unclear what recent instruments are attempting to measure and if they rely on ‘expert’ rather than patient input.

The Cambridge Pulmonary Hypertension Outcome Review (CAMPHOR) was the first -specific outcome measure to assess the impact of PH and its treatment on HRQoL and QoL [11]. The measure consists of three different scales: a symptom scale assessing energy, breathlessness and mood (25-items; low score indicating minimal symptoms); activity limitations scale with a 3-point rating system (15-items; range 0 to 30, lower score indicating minimal activity limitation); and a QoL scale (25-items; lower score indicating better QoL). The conceptual basis of the QoL scale is the needs-based model of QoL [12]. The model argues that QoL is determined by the extent to which one’s basic human needs are satisfied [12]. QoL is reduced when few needs are satisfied.

The measure was developed and validated in a unique PH reference center in United Kingdom and has been adapted for use in many countries, ﻿providing a valuable tool to reflect the actual patient experience in each country [13–20]. However, a validated version of the CAMPHOR for use in Brazil has been previously unavailable.

This report describes the adaptation and psychometric evaluation of the CAMPHOR into Brazilian Portuguese. It will allow the use of CAMPHOR in clinical practice as well as in global and local clinical trials involving Brazilian Portuguese-speaking patients with PAH and CTEPH.

## Methods

The translation and validation of the CAMPHOR was performed in 3 stages. Briefly, stage 1 involved translation into Brazilian Portuguese using the dual-panel methodology [12, 21]. In this approach, quality is checked at each stage of the process rather than being checked a posteriori. This same method has been used in all language adaptations of the CAMPHOR. The translation process emphasizes the importance of achieving conceptual equivalence allowing a valid comparison of CAMPHOR scores across countries.

The process consists of a bilingual panel followed by a lay panel. The role of the bilingual panel is to provide the initial translation of the questionnaire items and instructions into the target language. The translations are then assessed for comprehension of language by lay Brazilian people. Both panels were led by AROJr, fluent in both Portuguese and English, who encouraged the panel members to work as a team to decide on the most appropriate translation(s) for the questionnaire instructions and items. A representative from the team that developed the UK English questionnaire attended the panel. The role of this individual was to ensure that the precise conceptual meaning of the items was conveyed to the panel members. PH patients were not invited to take part in either panel as the sole purpose of the process was to determine the most appropriate wording for the questionnaire, rather than to comment on the appropriateness of the items.

Stage 2 involved testing the face and content validity of the translated questionnaire with a small group of PAH and CTEPH patients. Respondents were asked to complete the questionnaire and provide comments on the instructions, questionnaire items, and response format. Interviewees were also asked about specific items that had been highlighted as possibly problematic during the translation panels. In stage 3, the psychometric and scaling properties of the questionnaire were evaluated in a large cohort of PH (Group 1) and CTEPH (Group 4) patients. The CAMPHOR was administered on two occasions (time 1 and time 2), with 14 days between administrations. At both administrations, participants answered demographic questions (age, gender, marital status, employment), and rated their disease-severity as well as their general health. At the first administration only, participants also completed a comparator questionnaire, the Nottingham Health Profile (NHP). The NHP measures subjective health status and consists of six sections (energy level, pain, emotional reactions, sleep, social isolation and physical mobility).

### Patients and ethics

Patients were invited to participate if they were aged at least 18 years, were Brazilian speakers, and met the definition of PAH or CTEPH [22].

Patients were recruited from two PH reference centers in Brazil, Outpatient Clinic of Vascular Pulmonary Diseases of Hospital das Clínicas, Federal University of Minas Gerais, Belo Horizonte, and Hospital das Clínicas of University of Campinas, São Paulo. The study was approved by the Independent Ethics Committee of both Institutions (**CAAE:** 85372018.0.1001.5149 and **CAAE:** 85372018.0.2001.5404, respectively), and written informed consent was obtained from all participants before enrolment in the study.

### Statistical analyses

For stage 3, responses to the completed questionnaires were entered into an electronic database. The analyses performed are given below:

The distributional properties of the measure were explored through descriptive statistics (mean, standard deviation [SD], median and interquartile range [IQR]) and floor and ceiling effects (% of patients scoring the minimum and maximum possible scores, respectively). CAMPHOR scores were also compared by age group (above median versus below median) and gender.

Internal consistency was assessed using Cronbach’s alpha coefficients. Alpha measures the extent to which the items in a scale are inter-related. A low alpha (below 0.7) indicates that the items do not work together to form a scale.

The test-retest reliability of a measure is an estimate of its reproducibility over time when no change in condition has taken place. It was assessed by correlating scores on the scales obtained on two different occasions and assuming that no change has occurred to the patients between the administrations. A high correlation indicates that the instrument produces low random measurement error. A minimum value of 0.85 is required^4^. Test-retest reliability was assessed using Spearman’s rank correlation coefficients.

Convergent validity was determined by assessing the level of association between scores on one scale and those on a comparator scale that measures the same or related constructs.

Known group validity was assessed by testing the ability of the measure to distinguish between groups of patients that differ according to some known factor, considered likely to affect scores on the measure. The factors used for the present investigation were patient-perceived severity of disease (Mild / Moderate / Quite severe / Very severe) and patient-perceived general health (Very good / Good / Fair / Poor). Non-parametric statistical tests for independent samples (Mann-Whitney U Test for two groups) were employed to test for differences in CAMPHOR scores between groups.

All analyses were conducted using SPSS (version 25.0).

## Results

The bilingual panel (stage 1) included six individuals (4 females, 36.67 ± 19.97 years old), whose first language was Brazilian Portuguese and who were fluent in English. Whenever there was a divergence between members of the panel, a final decision was taken after hearing the decision of the whole group of translators. The translation obtained by this panel meeting was able to capture the same concepts as the English questionnaire and produced an acceptable and comprehensive formulation of the concept.

The lay panel included other six Brazilian-Portuguese individual (4 females) who did not speak English. The panel members were representative of a range of ages (51.17 ± 14.91) professions and gender. Those who participated in this panel had an average to lower than average educational level and were considered as typical of the target population. Participants were presented with the translations provided by the bilingual panel and were asked to comment on whether the phrasing and choice of words was acceptable or whether any changes were required to improve comprehension. When changes were deemed as necessary, the professor (AROJr) always went back to the original questionnaire to ensure that the meaning had not been changed as well as checked with the UK representative. This panel was able to guarantee the most appropriate translation for lay individuals without compromising the original meaning.

The field test interviews (stage 2) were performed with 12 Brazilian PAH patients (6 females, 54.98 ± 11.99 years old), with disease severity ranging from mild to very severe. All patients agreed that the questionnaire was relevant, acceptable and meaningful. The interviewees were asked to comment on items that had been highlighted a priori as potentially problematic for some respondents, thus contributing to the final wording of the instrument. Importantly, for some few sentences, the interviewees were asked for making an option in between previous suggestions coming from the panels.

The psychometric evaluation (stage 3) was performed with one hundred and two patients. All included patients who had been adequately diagnosed with PAH or CTEPH, according to current international PAH guidelines [22]. Demographic information is presented in Table 1. Most respondents were female and married or living as married. Over half of the sample reported being retired or on long-term sick leave/retired due to disability. Disease information is shown in Table 2. Most patients rated their disease severity as ‘moderate’ or ‘quite severe’ and their general health as ‘good’ or ‘fair’. Table 3 shows descriptive statistics for the questionnaires at both time points. High floor effects were observed for most of the NHP scales.

**Table 1:** Demographic and disease information (*n* = 102)

**Age, years**		
Mean (SD)	48.8 (14.5)	
Range	24.11–86.83	
**Gender**	**n**	**%**
Male	20	19.6
Female	82	80.4
**Marital Status**		
Married/Living as Married	66	64.7
Divorced	7	6.9
Widowed	5	4.9
Single	23	22.5
**Work Status**		
Full-time	16	15.7
Part-time	3	2.9
Retired	22	21.6
Long-term sick leave/retired due to disability	29	28.4
Homemaker	17	16.7
Unemployed	8	7.8
Other	6	5.9

**Table 2:** Disease Information at Time 1 (*n* = 102)

**Patient-perceived disease severity (%)**	
Mild	13 (12.7)
Moderate	36 (35.3)
Quite Severe	36 (35.3)
Very Severe	16 (15.7)
**Patient-perceived general health (%)**	
Very Good	4 (3.9)
Good	38 (37.3)
Fair	38 (37.3)
Poor	21 (20.6)
**Receiving treatment (%)**	
Yes	95 (93.1)
No	5 (4.9)
**Disease Duration, years**	
Mean (SD)	7.6 (6.3)
Range	0.33–44

**Table 3:** Questionnaire descriptive statistics

	n	Median (IQR)	Min - Max	% scoring minimum	% scoring maximum
**Time 1**					
CAMPHOR Symptoms	100	10 (6–17)	0–25	1	1
CAMPHOR Activities	101	10 (6–14)	0–24	3.9	0
CAMPHOR QoL	100	8 (4–14.8)	0–25	2.9	2
**NHP**					
Energy Scale	101	33.3 (0–66.7)	0–100	33.3	18.6
Pain Scale	100	12.5 (0–37.5)	0–100	35.3	1
Emotional Reactions	101	22.2 (11.1–66.7)	0–100	18.6	3.9
Sleep Scale	102	20 (0–80)	0–100	42.2	7.8
Social Isolation	102	0 (0–40)	0–100	57.8	6.9
Physical Mobility	101	37.5 (12.5–62.5)	0–87.5	10.8	0
**Time 2**					
CAMPHOR Symptoms	93	10 (5.5–18)	0–25	3.9	2
CAMPHOR Activities	94	10 (6–15)	0–27	2	0
CAMPHOR QoL	93	7 (3–15)	0–25	2.9	3.9

The internal consistency and reproducibility of the CAMPHOR is shown in Table 4. Cronbach’s alpha coefficients were above 0.9 on all three CAMPHOR scales, indicating good internal consistency. The CAMPHOR showed excellent test-retest reliability, achieving Spearman’s rank correlation coefficients above 0.85 on all scales.

**Table 4:** Internal consistency and reproducibility

	Internal consistency (Time 1)	Test-retest reliability
CAMPHOR Symptoms	0.92	0.88
CAMPHOR Activities	0.92	0.87
CAMPHOR QoL	0.92	0.92

Table 5 shows the correlations between scores on the CAMPHOR scales and scores on the NHP sections at Time 1. The CAMPHOR Symptoms scale and Activities scale correlated most highly with the Physical Mobility section of the NHP. The CAMPHOR QoL scale was most strongly associated with scores on the Emotional Reactions and Social Isolation sections of the NHP, indicating the importance of these factors to QoL in PH. Moderate correlations were observed with scores on the remaining NHP sections.

**Table 5:** Correlation coefficients between CAMPHOR scales and NHP scales

	Symptoms	Activities	QoL
**NHP**			
Energy Scale	0.77	0.67	0.61
Pain Scale	0.53	0.45	0.46
Emotional Reactions	0.72	0.59	0.83
Sleep Scale	0.42	0.40	0.52
Social Isolation	0.45	0.38	0.70
Physical Mobility	0.83	0.82	0.69

Note: All correlations significant at the 0.01 level (2-tailed) except where marked.

Table 6 shows CAMPHOR scores for patients grouped by gender and age group (above versus below median age). No significant differences in scores were found between older and younger patients on any of the CAMPHOR scales.

**Table 6:** CAMPHOR scores by gender and age group

		Symptoms		Activities		QoL
	n	Median (IQR)	n	Median (IQR)	n	Median (IQR)
**Gender**						
Male	20	7 (3.3–10)	20	8 (4.3–10)	20	6.5 (3.3–8)
Female	80	12.5 (7–17)	81	11 (6–16)	80	8.5 (4.3–16.8)
***p***		< 0.01		< 0.05		< 0.05
**Age**						
Below Median	50	10.5 (5.8–16.3)	51	10 (6–14)	51	10 (6–15)
Above Median	50	10 (6–17)	50	9.5 (5.8–16.3)	49	7 (3–13.5)
***p***		0.64		0.65		0.13

There was a significant difference between males and females on all CAMPHOR scales, with females scoring higher than males. A significant association was found between gender and perceived general health (χ^2^ (101) = 4.5, *p* < 0.05). Most males perceived their general health to be very good/good, whereas most females rated their general health as fair/poor.

**Fig. 1:**
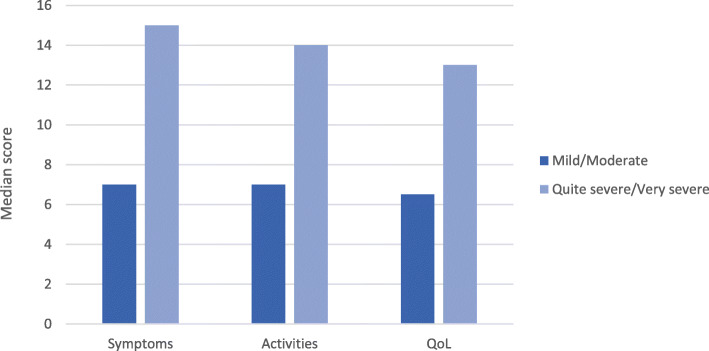
Median CAMPHOR scale scores by patient-perceived disease severity. Note: All comparisons significant at the 0.001 level (2-tailed)

**Fig. 2:**
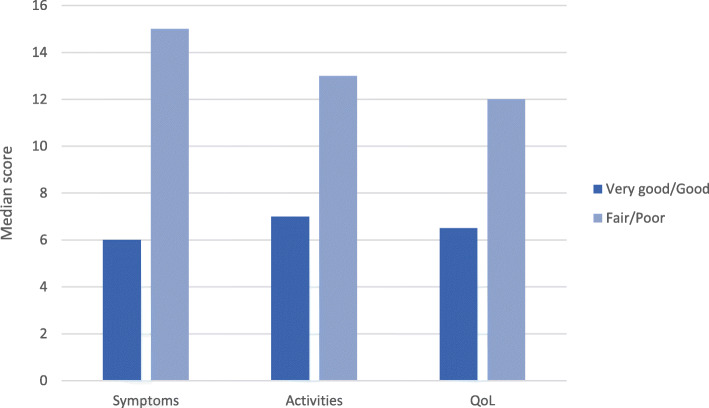
Median CAMPHOR scale scores by patient-perceived general health. Note: All comparisons significant at the 0.001 level (2-tailed)

Results of the known group analyses are shown for patient-perceived disease severity (Fig. 1) and patient-perceived general health (Fig. 2). For disease severity, the categories were grouped into ‘mild/moderate’ and ‘quite severe/very severe’. The categories for general health were grouped according to ‘very good/good’ and ‘fair/poor’. These groupings were selected due to few participants reporting at the extreme ends of disease severity and general health questions. There were significant differences in CAMPHOR scale scores between patients who differed according to their perceived disease severity and general health. Patients who rated their disease severity as ‘Quite severe/Very severe’ had significantly worse scores on all CAMPHOR scales than patients who rated their disease severity as ‘Mild/Moderate’ (*p* < 0.001). Also, patients who considered their general health to be ‘Fair/Poor’ had worse scores on all CAMPHOR scales than patients who rated their health as ‘Very good/Good’ (*p* < 0.001).

## Discussion

The aim of the present study was to adapt and validate the CAMPHOR questionnaire into Brazilian Portuguese [9]. The results indicated a successful adaptation of the CAMPHOR for use with Portuguese speaking patients in Brazil. The translation process resulted in a Brazilian-Portuguese version that was expressed in common, natural language and matched the original UK version in terms of conceptual equivalence. All patients who participated in the cognitive debriefing interviews reported that the translated questionnaire was easy to complete and that it covered important aspects of their experience of living with PH.

As PH, as a whole, is a progressive chronic condition, the evaluation of HRQoL is an important component of outcomes both in clinical trials and in clinical practice [10, 23, 24]. Generic measures, such as the NHP and SF-36 have been commonly used to evaluate HRQL in PH. However, they are unable to capture all areas of concern to this patient population [25]. The more specific the instruments are for this evaluation, the better their ability to capture minimal change associated with effective treatment [6–8].

In the current era of more individualized management of PH patients, combination therapy has become the standard approach for most patients as soon as they are diagnosed and have their clinical status stratified [26]. A recent report [27] ﻿has shown a therapeutic effect on HRQoL over time in newly diagnosed PAH patients treated with sequential combination, reinforcing the importance of QoL as an important aspect to take into consideration in a holistic evaluation of PH patients**.** The most commonly used criteria for guiding therapeutic clinical decision making are severity of disability (WHO FC), 6MWD, hemodynamic variables and biomarkers, among others [27]. It is noteworthy to mention that CAMPHOR was the first disease-specific instrument developed to evaluate the impact of PH on HRQoL and needs-based QoL [11].

Interestingly, high floor effects were observed for most of the NHP scales, indicating that this measure is not well targeted to PH patients, while this was not observed with the CAMPHOR scales. The CAMPHOR demonstrated excellent internal consistency and test-retest reliability, indicating that the items work together to form a scale as well as a low random measurement error, respectively. Investigation of the correlations between scores on the CAMPHOR and NHP scales provided evidence of convergent validity, suggesting that the two measures of these constructs that theoretically should be related, are in fact related.

As expected, scores on the NHP Physical Mobility scale correlated highly with scores on the Symptoms and Activities scales of the CAMPHOR. This was not surprising considering physical mobility is an indicator of functioning and will be influenced by the presence of symptoms. Scores on the QoL scale showed at least moderate correlations with scores on the NHP scales, suggesting that multiple factors influence QoL in PAH and CTEPH patients.

No significant differences in scores were found between older and younger patients on any of the CAMPHOR scales. However, significant differences in scores were found between males and females on all CAMPHOR scales, with females scoring higher than males. A chi^2^ test of independence was performed to assess the relation between gender and perceived general health. This revealed that most males rated their general health as ‘very good/good’ in contrast to most females who rated their general health as ‘fair/poor’. It is likely that these differences contributed to females reporting worse symptoms, functioning and QoL ion the CAMPHOR.

In the present study, there were few participants reporting at the extreme ends of disease severity and general health questions. As in other CAMPHOR validation reports, this finding does not seem to interfere with the results of the validation since this research was not designed to verify the impact of the disease on the HRQoL of PH patients. Notwithstanding, evidence of known group validity was demonstrated by the ability of the CAMPHOR to distinguish between subsets of patients with distinct perceptions of disease severity and general health. Thus, participants who rated their disease as more severe (i.e., Quite severe/Very severe) had significantly worse scores on all CAMPHOR scales than patients who rated their disease as less severe (Mild/Moderate), and patients who considered their general health to be ‘Fair/Poor’ had worse scores on all CAMPHOR scales than patients who rated their health as ‘Very good/Good’. These results demonstrate the ability of the Brazilian Portuguese CAMPHOR to detect meaningful differences between patients.

In conclusion, the Brazilian version of the CAMPHOR questionnaire demonstrated good psychometric properties. ﻿This new instrument has proved to be an accurate and reliable instrument for assessing both HRQL and QoL in Brazilian PH patients. It is expected that this new version of CAMPHOR will prove to be a valuable tool to evaluate patients both in clinical practice and in future research involving Brazilian PH patients.

## Data Availability

Data can be accessed upon request to corresponding author.

## Abbreviations

PH: Pulmonary Hypertension
HRQoL: Health-related quality of life
CAMPHOR: Cambridge Pulmonary Hypertension Outcome Review
NHP: Nottingham Health Profile
CPTEH: Chronic thromboembolic pulmonary hypertension
WHO FC: World Health Organization functional class
6MWT: Six-minute walk test
BNP and NT-proBNP: Biomarkers of cardiac function
